# Intraoperative Autologous Adipose-Derived Therapies and PRP as Add-On in the Surgical Treatment of Cryptoglandular and Crohn’s Disease-Related Perianal Fistula—A Systematic Review

**DOI:** 10.3390/bioengineering13040393

**Published:** 2026-03-28

**Authors:** Merel M. Verweij, Mustafa Uguten, Michiel T. J. Bak, Caroline D. M. Witjes, Annemarie C. de Vries, Ilse Molendijk, Joris A. van Dongen, Oddeke van Ruler

**Affiliations:** 1 Department of Gastroenterology and Hepatology, Erasmus Medical Center, University Medical Center Rotterdam, 3015 GD Rotterdam, The Netherlands; 2 Department of Plastic, Reconstructive and Hand Surgery, Medical Center Leeuwarden, 8934 AD Leeuwarden, The Netherlands; 3 Department of Plastic, Reconstructive and Hand Surgery, University Medical Center Utrecht, University of Utrecht, 3584 CX Utrecht, The Netherlands; 4 Department of Surgery, IJsselland Hospital, 2906 ZC Capelle aan den IJssel, The Netherlands

**Keywords:** perianal fistula, cryptoglandular, Crohn’s disease, platelet-rich plasma, adipose-derived therapy, regenerative medicine

## Abstract

Background: The treatment of perianal fistulas remains challenging, with low healing and high recurrence rates. Autologous adipose-derived regenerative therapies and platelet-rich plasma (PRP) have emerged as adjuncts to surgical intervention for cryptoglandular and Crohn’s disease (CD)-related perianal fistulas (PAF). This systematic review evaluates the outcomes of these therapies as an add-on to surgical intervention. Methods: A systematic search was conducted in several online databases up to December 2025. Studies with ≥10 patients reporting on the use of intraoperative autologous adipose-derived therapies and/or PRP for the treatment of cryptoglandular or CD-related PAF, and clinical healing rates, were included. Other outcomes comprised radiologic healing (as defined in the study), recurrence rates and complications. The study quality was assessed with the Effective Public Health Practice Tool. Results: In total, 28 studies on individual cases were included (*n* = 1017 patients, range 10–219) (17 in cryptoglandular PAF, 8 in CD-related PAF and 3 in both entities). A total of 57% of the studies were rated low quality. In cryptoglandular PAF, reported healing rates with adipose-derived therapies ranged from 50% to 90% across studies of low to good methodological quality. For PRP, three of the four randomized trials demonstrated no superiority over standard care. In CD-related PAF, healing rates after treatment with adipose-derived therapies ranged from 40% to 80%. For PRP, three studies, of which two were low quality, reported highly variable healing rates (33–80%). Radiologic healing, reported in 10 studies, ranged from 38 to 76% in cryptoglandular and 33–75% in CD-related PAF. Recurrence rates remained <17% for adipose-derived therapies and <31% following treatment with PRP. Major complications occurred in <15% of the patients. Conclusions: High heterogeneity with regard to fistula complexities, outcome definitions and surgical method was observed in the available studies on autologous add-on therapies. This hinders an overall effectiveness analysis. The promising healing rates, low recurrence rates after healing and low complication rates warrant high-quality trials.

## 1. Introduction

A perianal fistula is an abnormal tract connecting the rectum or anal canal to the perianal skin [1]. The majority (90%) originate from a cryptoglandular source. In addition to this origin, perianal fistulae are highly prevalent in patients with Crohn’s disease (CD) as approximately 25% of patients with CD develop a perianal fistula within 20 years of their disease diagnosis [2]. These fistulae cause disabling symptoms including fluid or pus discharge, perianal pain and fecal incontinence, and diminishing the quality of life [3].

To control symptoms and perianal sepsis, seton placement and antibiotics are often initiated. For cryptoglandular perianal fistula (PAF), surgical closure remains the cornerstone of treatment, with transanal flap repair (TAFR) and ligation of the intersphincteric tract (LIFT) being the most commonly used techniques. However, reported healing rates are inconsistent and modest, ranging from 58 to 80%, with high recurrence rates of 20–52% [4,5,6]. In CD-related PAF, a combined medical and surgical approach is preferred to achieve fistula closure including anti-tumor necrosis factor (anti-TNF) therapy along with surgical treatment in surgically amenable patients [7,8]. However, the majority of the CD-related PAFs are complex (involvement of the anal sphincter, multiple branches, extensions and/or, internal or external openings) and to date long-term remission is only achieved in 37% of patients [9]. Residual or recurrent disease often necessitates repeated interventions, radiological assessments and hospital admissions. In refractory cases, diversion by ostomy or even proctectomy may be required, leading to substantial disease burden, medical costs and productivity loss due to impaired ability to work [10,11]. Consequently, new therapies to improve outcomes after fistula surgery for both cryptoglandular and CD-related PAF are urgently needed.

Mesenchymal stromal cells (MSCs) and platelet-rich plasma (PRP) have emerged as promising add-ons to fistula surgery. MSCs stimulate wound healing by releasing paracrine factors to stimulate tissue regeneration, modulate inflammation and angiogenesis [12,13]. These stromal cells can be harvested from bone marrow or adipose tissue. Adipose tissue can be easily harvested by liposuction and directly applied as fat grafts, stromal vascular fraction (SVF) or adipose-derived stromal cells (ASCs). PRP has been studied for its enhanced wound healing effect due to the high concentration of growth factors [14,15].

Adipose-derived therapies can be either allogeneic or autologous. Allogeneic (donor) or cultured autologous approaches require harvesting, laboratory cell expansion and secondary administration, resulting in logistic challenges and higher medical costs. Additionally, allogeneic cells may be immunogenic, with a possible graft-versus-host risk, and laboratory manipulation prior to administration may influence therapeutic effects because cells change during culture in terms of function and phenotype. Following the withdrawal of the only commercially available allogeneic MSCs from the market, interest has shifted toward investigating the effects of autologous therapies, with the advantages of direct applicability and low costs [16].

Since various therapeutic approaches and heterogeneous patient groups are used in research settings, it remains challenging to compare outcomes and assess generalizability. Therefore, we aimed to evaluate the use and efficacy of directly applicable (non-cultured) autologous adipose-derived regenerative therapies and PRP in both cryptoglandular and CD-related PAF.

## 2. Materials and Methods

### 2.1. Search Strategy

This systematic review was registered in PROSPERO [CRD42024569313] and followed the Preferred Reporting Items for Systematic Reviews and Meta-analyses (PRISMA) statement [17]. An extensive literature search was performed in Embase, Ovid, Web of Science Core Collection and CINAHL in collaboration with the Medical School Library of the Erasmus Medical Center until 2 December 2025. The following keywords were used “perianal fistula”, “autologous adipose-derived therapies” and “blood derived therapies”. The full search string is provided in Supplementary File S1.

### 2.2. Eligibility Criteria

Studies reporting on (I) the use of intraoperative harvested non-cultured autologous adipose-derived therapy and/or PRP as an add-on to surgical intervention for the treatment of cryptoglandular or CD-related PAF and (II) the healing rates of the perianal fistula after intervention with adipose-derived therapy or PRP were included. The eligible types of therapy were fat grafting or similar treatments (lipografting, lipofilling, Colemann fat or similar derivates as macrofat, microfat or nanofat; further defined as fat grafting), stromal vascular fraction (SVF) or adipose-derived stem cells (ASCs) and blood plasma, platelet-rich plasma (PRP), platelet-rich fibrin (PRF) and all combinations of the aforementioned therapies. Non-eligible studies were animal or in vitro studies, case-reports, case-series describing less than ten patients, reviews, conference abstracts and studies from the same research group with overlapping patients. In case the included patient population was overlapping, the study with the largest cohort size or longest follow-up period was included. A snowball approach was applied across all included studies.

### 2.3. Data Extraction

Data were independently extracted by the two reviewers (M.M.V. and M.U.) by using Covidence. Collected study characteristics comprised: first author, year of publication, study design, sample size, intervention and control groups (if applicable). Subsequently, patient-related factors were collected including sex, age, BMI, smoking, comorbidities, fistula anatomy (e.g., intrasphincteric, transsphincteric) and extent (side branches, number of internal and external openings), prior interventions and concomitant medication (biologicals or immunomodulators) for CD-related PAF. The following intervention-related characteristics were collected: type of add-on therapy, harvesting site and technique (e.g., the abdomen with Coleman technique), infiltration site and manner (e.g., infiltration along the fistula tract), injected volume, sample characterization (cell viability), and preoperative and postoperative medical treatment (e.g., antibiotics, analgesics, anti-TNF). Finally, we collected data on the reported clinical healing rates of the intervention as defined in the study. In addition, data were collected including radiologic healing (as defined in the study), recurrence rates, reinterventions, complications defined as minor (hematoma, proctalgia, local pain at harvesting site) and major (adverse events necessitating intervention, infections, abscesses, ostomy placement or necessity for proctectomy), incontinence scores (e.g., Wexner or Vaizey), pain and quality of life (QoL), if reported.

### 2.4. Risk of Bias Assessment

The study quality was assessed by the two independent reviewers according to the Effective Public Health Practice Project (EPHPP) [18]. With this tool the quality of different study designs can be assessed, consisting of different domains: design, selection bias, confounders, data blinding, data collection and dropouts. Studies were scored as ‘strong, ‘moderate’ or ‘weak’. The risk of bias was assessed in randomized controlled trials (RCT’s) with the Cochrane risk of bias tool (RoB2). Conflicts were resolved between the reviewers.

## 3. Results

### 3.1. Study Selection, Design and Quality

The search yielded 885 studies. After removal of duplicates, screening for eligibility and cross-reference checks, 58 studies were assessed in full text. In total, 28 studies were included [Supplementary Table S1] [19,20,21,22,23,24,25,26,27,28,29,30,31,32,33,34,35,36,37,38,39,40,41,42,43,44,45]. Five (18%) studies were RCT’s [19,23,30,34,42], twelve (43%) studies concerned single group cohort studies [22,24,25,27,28,32,36,37,41,43,44,45], two (7%) two-group cohort studies [29,35], three (11%) prospective studies [31,38,46], and six (21%) retrospective studies [20,21,26,29,33,39]. Sixteen (57%) studies were assessed as weak [21,26,28,29,30,31,32,33,35,36,37,38,39,40,43,44,45]. Six studies (21%) were assessed as moderate [20,25,27,40,41]. And another six studies (21%) were rated strong [19,22,23,24,34,42] [Supplementary Table S2a]. Three RCT’s were considered low risk of bias and in the other two there were some concerns [Supplementary Table S2b]. Seventeen studies (61%) concerned cryptoglandular PAF [19,20,21,23,24,26,29,30,33,34,35,36,38,39,41,42,46], eight studies (29%) focused on CD-related PAF [19,22,27,28,37,44,45,47], and three studies (11%) included both entities [31,32,48].

### 3.2. Patient and Fistula Characteristics

In total, 1017 patients were treated with autologous adipose-derived therapies (fat grafting, SVF or ASCs) (*n* = 442, 43%) or intraoperative PRP (*n* = 575, 57%) (range 10–219) [Table 1]. The mean age ranged from 31 to 58 years, and 37% of the included patients were female (range 9–75%). The follow-up time varied between 6 and 77 months. Active smoking status was assessed in half of the studies (50%) reporting percentages from 16% to 56.3% [21,22,23,26,27,28,34,37,38,40,42,43,45,47]. Complexity of the fistula was assessed in 16 studies (57%), of which 12 studies (43%) reported complex fistulas with side branches in 10% to 62% of patients [19,20,22,24,28,31,37,41,43,44,45,46,47]. Four of these studies (14%) reported horseshoe fistulas in 3.5% to 38% of patients [19,34,40,45]. Three studies (11%) only included patients with a simple single-tract fistula [21,35,42]. Previous surgical interventions were reported in 20 studies (71%) ranging from 30% to 100%. Among studies including CD-related PAF (*n* = 11), nine (82%) reported concomitant use of anti-TNF or other biological therapy in 48% up to 100% of patients [22,25,27,28,32,37,40,44,45,47] [Table 1].

### 3.3. Treatment Characteristics

In total, thirteen studies (46%) administered fat grafts, SVF, or ASCs in cryptoglandular (*n* = 6) (21%), CD-related PAF (*n* = 5) (18%) and in both (*n* = 2) (7%) [19,21,25,31,32,33,35,37,38,39,40]. A combination of PRP and SVF was applied in two studies (7%) in the treatment of cryptoglandular (*n* = 1) (4%) and CD-related PAF (*n* = 1) (4%) [41,45]. For adipose-derived therapies, the abdomen was the most common donor site for fat harvesting (77%) [19,21,25,32,33,35,37,38,39,40]. Only four studies (31%) reported on characteristics of the add-on therapy (mostly cell yield or viability) of the adipose-derived therapies [25,28,31,37]. Thirteen studies (46%) investigated the use of PRP for the treatment of perianal fistulas, in cryptoglandular PAF (*n* = 10) (29%), in CD-related PAF (*n* = 2) (11%) and in both entities (*n* = 1) (4%) [20,22,23,24,26,27,29,30,34,36,42,43,46].

PRP was obtained either by centrifugation (*n* = 9) (69%) [20,22,23,24,29,30,36,42,46] or by the gravitational platelet separation (*n* = 3) (23%) [26,27,34]. One study applied “Obsidian^®^ regenerative fistula treatment” an autologous, platelet-rich bioactive matrix derived from the patient’s own blood [43]. No studies on PRP reported on characterization of the obtained sample.

Almost all of the included studies (24/28, 86%), injected the adipose-derived therapy and/or PRP into the fistula wall from the internal orifice to the external opening [Supplementary Table S3]. The remaining four studies (14%) administered the product by filling the fistula tract [26,27,33,43]. In the vast majority of studies (*n* = 27) (97%), the therapy was administered as addition to surgical intervention; one study reported on repeated injections of regenerative therapy without any surgical intervention [30]. The surgical interventions included curettage of the fistula tract with closure of the internal opening (IO) (*n* = 21) (75%) [21,22,23,24,25,27,28,29,31,32,33,35,36,37,38,39,43,44,45,46,47], TAFR (*n* = 4) (14%) [19,20,26,41] or LIFT (*n* = 2) (7%) [34,42].

The majority of the included studies (*n* = 19) (68%) reported on clinical healing as “absence of discharge and closure or epithelization of the external opening”. Three studies (11%) defined their endpoint as the absence of discharge [29,31,43]. The remaining six studies (21%) did not report on an outcome definition [27,28,30,35,37,38].

### 3.4. Cryptoglandular Perianal Fistulae

#### 3.4.1. Clinical Healing Rates in Cryptoglandular Perianal Fistulae

##### The Use of Adipose-Derived Therapies

Eight studies investigated the use of fat grafting (*n* = 3), SVF (*n* = 3), ASCs (*n* = 1) or platelet-rich stroma (PRS) (*n* = 1) in cryptoglandular fistulae. Study designs comprised one RCT (4%), four prospective cohorts (14%), and three retrospective cohorts (11%) [19,21,31,33,35,38,39,41]. In the RCT of Ascanelli et al., ASCs as an add-on to surgical intervention (fistulectomy, VAAFT, TAFR or closure of the internal opening) were compared with surgical intervention alone in a patient group in which 59% underwent prior surgery; however, the extent or branches of the fistula were not reported. Significant higher healing rates were reported at 4 weeks (64% vs. 15.5%, *p* < 0.001) and 3 months (88% vs. 64%, *p* < 0.001) in the ASC group. However, no difference in clinical healing was observed at six months postoperatively (86% vs. 81%, *p* = 0.477). Recurrence rates were comparable between groups (3% vs. 0%, *p* = 0.49) at 12-month follow-up including reinterventions in both groups (14% vs. 14%) [19]. From the observational studies, two studies investigated the use of fat grafting combined with curettage and closure of the internal opening. In the study by Huang et al., about one-third had recurrent fistula disease at inclusion, and healing rates of 71% at 3 months were reported; reinterventions were planned in 33% of the patients [32]. In contrast, the study by Stroumza et al. included a recurrent patient group, and reported healing rates of 73%, 6 months postoperatively. Neither reported on recurrence rates [38]. Three cohort studies assessed SVF with curettage and closure in patients with mostly one single tract, reporting healing rates of 69–86% at 3–6 months; reinterventions and recurrence was either not reported (*n* = 2) or not observed (*n* = 1) [31,33,35]. The study combining SVF and PRP (PRS) in a refractory patient group, achieved fistula healing in 84% at 1-year follow-up and 77% at a median follow-up of 3 years, including reinterventions in 10%. A recurrence rate of 9% was observed in a median follow-up of 4 years [41] [Table 2].

##### The Use of Platelet-Rich Plasma (PRP)

Eleven studies (39%) reported on the use of PRP in cryptoglandular PAF of which four were RCTs (14%), five one-group cohort studies (18%), one retrospective cohort (4%), and one retrospective comparative cohort study (4%) [20,23,24,26,29,30,34,36,42,43,46]. Overall, healing rates ranged between 38% and 100% (median follow-up at 12 month). The randomized trial of Madbouly et al. demonstrated a significant improvement in healing when PRP was combined with LIFT compared to LIFT alone (78% vs. 59%, *p* = 0.03) at four-weeks follow-up. However, only 4% of the patients had undergone previous fistula surgery and fewer than 10% presented with secondary tracts or a horseshoe fistula, indicating a relatively simple patient group. In both groups, 10% recurred at one-year follow-up [34]. In the remaining three RCTs, no significant differences in healing were observed between PRP and control groups [23,30,42]. One of these randomized trials compared PRP with curettage and closure of the IO to fibrin glue in a patient population in which about 50% had multiple tracts, reporting no significant differences at 12-month follow-up (48% vs. 42%, *p* = 0.608) [23]. Bananzadeh et al. included patients with only one fistula tract and compared PRP with LIFT to LIFT alone reporting no differences at 12-month follow-up (90% vs. 78%, *p* = 0.153) [42]. The trial of Hermann et al. compared repeating PRP injections only, without any additional surgical intervention, to TAFR in a population with a single fistula tract. Despite the numerical difference (71% vs. 58%), no statistical difference in clinical healing at 12 months postoperatively was observed (*p* = 0.152) [30]. The study by Bak et al. (*n* = 219) compared PRP with TAFR to TAFR alone in a complex patient population, 62% had multiple tracts and 96% underwent prior surgery. Using inverse probability of treatment weighting, no difference in clinical healing at 12-month follow-up was found (74% vs. 77%, *p* = 0.71) [20]. Reinterventions were in 8% in both groups. Recurrence rates did not differ significantly between intervention (0%–31%) and control groups (0%-33%) across all five studies, with follow-up ranging from one year up to a mean of 7 years [Table 2].

##### Radiologic Healing

Radiologic healing was assessed in four studies in cryptoglandular PAF, three after treatment with adipose-derived therapy and one after PRP. Two studies assessed combined closure as the primary outcome, although neither reported on a definition. Stroumza et al. reported a 73% healing rate at 6-mmomonthollow-up and Hermann et al. (PRP) reported a combined healing rate of 64% at 2-month FU. Across all studies, combined clinical and radiologic healing ranged from 38 to 76%, assessed between 2- and 12-month follow-up [19,29,38,41].

#### 3.4.2. Complications

Postoperative complications were reported in 25 studies (89%) [19,20,21,22,23,24,25,26,27,28,30,31,32,33,34,35,36,37,38,39,40,41,42,46,49]. For cryptoglandular PAF, minor complications comprised hematoma or pain at liposuction site in 2–21%, proctalgia in up to 18% and conservatively treated bleedings in a maximum of 4% [19,20,22,23,35,39]. In the study by de la Portilla et al., temporary fecal urgency and incontinence were both observed in 3% [24]. Major complications including postoperative perianal abscesses and/or postoperative bleeding requiring interventions occurred in 2–4% of patients receiving the add-on therapy and in up to 12% of those undergoing surgery alone [19,20,43]. One patient, treated with PRP, developed a scrotal and prostate abscess (0.1%) [24].

#### 3.4.3. Quality of Life (QoL)

Quality of life was assessed in two studies including patients with cryptoglandular PAF. Madbouly et al. compared QoL between PRP with LIFT to LIFT only at 4-weeks follow-up and reported a significant improvement in QoL in favor of the PRP and LIFt group (*p* < 0.05). However, the study did not specify the method used to assess this outcome [34]. De la Portilla et al. compared QoL, measured with the Short Form Health Survey, at baseline and 12-month follow-up in patients treated with PRP; no differences in QoL were observed [23].

### 3.5. Crohn’s Disease-Related Perianal Fistulae

#### 3.5.1. Clinical Healing Rates in Crohn’s Disease-Related Perianal Fistulae

##### The Use of Adipose Tissue

Eight studies investigated the use of fat grafting (*n* = 3), SVF (*n* = 4), or PRS (*n* = 1) for CD-related perianal fistulae; all were single-group observational cohort studies [25,31,32,37,40,44,45,50]. Four studies assessed SVF as an add-on to curettage and closure of the internal orifice [28,31,37,44]. Healing rates varied substantially between studies (40%, 75% and 80% at 6-mmonthfollow-up in three studies and 60% at 12-month follow-up in one study). While Sorensen et al. and Potenza et al. reported relatively high clinical healing rates of 75% and 80%, these studies did not specify how healing was defined and largely included patients with a single fistula tract. A total of 10% (*n* = 1) underwent a reintervention in the study by Potenza et al. Recurrences were reported in 25% (*n* = 3) and 10% (*n* = 1) at 12 months. In contrast, Guillo et al. observed a 60% healing rate in a cohort where the majority of patients presented with multiple fistula tracts, indicating a more complex patient population. Recurrence after healing was observed in 10% (*n* = 1) after 3-years follow-up. Three studies reported on the use of fat grafting combined with surgery resulting in clinical healing rates ranging from 43 to 67% at 3–6 months [25,32,40]. The study by Laureti et al. assessed on a long-term follow-up and reported overall healing in 87% and recurrence in 7% at 7 years, with up to 2 reinterventions in 20% [40]. Treatment with platelet-rich stroma (PRS, a combination of PRP and SVF) achieved healing in 52% at one year and in 88% at a mean follow-up of 3.7 years, including a median of 2 reinterventions in 52% of the patients [45]. Recurrence was reported in 8%. Both included a comparable complex patient group, with recurrent disease, and multiple branches [Table 2].

##### The Use of Platelet-Rich Plasma (PRP)

Three cohort studies evaluated PRP in CD-related PAF, with approximately 50% of patients presenting with recurrent disease in all studies [22,27,43]. Göttgens et al. assessed the outcomes of PRP with TAFR and reported fistula healing in 80% at 3-month follow-up with a 10% (*n* = 1) recurrence rate at 12 months [27]. The two remaining studies combined PRP with curettage and closure of the internal opening and reported healing rates of 33% and 38% at six months postoperatively [22,43]. Recurrence rates were not reported in both studies [Table 2].

#### 3.5.2. Radiologic Healing

Six studies including CD-related PAF assessed radiologic healing after treatment with adipose-derived therapies [25,28,37,40,44,45]. Of these, four studies assessed combined closure as their primary outcome. Two studies assessed at 6-month FU, reporting on 43% and 50% healing [25,44], whereas the other two studies with long-term FU (3 and 7 years) reported 70% and 87% healing [28,40].

#### 3.5.3. Complications

In CD-related PAF, minor complications related to fat harvesting including hematoma or pain at the liposuction site, ranged from 5 to 40%, and liposuction bleeding occurred in 5%. Proctalgia occurred in 7–19%, temporary fecal incontinence in up to 3% and perianal wound infection in up to 10% [25,28,32,40,45]. Rates of minor postoperative bleeding without the need for reintervention were reported in 3–8% [45]. Major complications included postoperative abscess requiring surgical intervention in up to 5% and a labia majora abscess after injection of adipose tissue was reported in one patient [25]. Despite complications at liposuction site, most complications were related to the surgical intervention itself rather than the add-on therapy.

#### 3.5.4. Quality of Life (QoL)

Three studies in CD-related PAF assessed quality of life as a secondary outcome. Potenza et al. reported a significant improvement in QoL, measured with the Cleveland Global Quality of Life, in healed patients treated with SVF at 6 month compared with baseline (*p* = 0.05) [44]. The remaining two studies observed improvements in QoL, assessed with the (Short) Inflammatory Bowel Disease-Questionnaire (IBDQ) in patients treated with fat grafting or SVF from baseline to 12-month follow-up, although these improvements did not reach statistical significance [28,40].

## 4. Discussion

In this systematic review, the current evidence on the use of intraoperative autologous regenerative therapy for cryptoglandular and CD-related PAF, specifically intraoperative adipose-derived therapies and PRP, was assessed. Although the efficacy of these therapies as adjuncts to surgery remains unclear due to substantial heterogeneity, both were associated with low recurrence rates and few and mostly minor complications. The included studies varied widely in patient populations, surgical techniques, add-on therapies, and outcome definitions. Many studies were limited by small sample sizes and short follow-up periods, further constraining the strength of evidence. The limited number of randomized trials hindered meaningful comparison between treatment strategies.

The rationale for using adipose-derived therapies is based on the ability of ASCs to support tissue repair mechanism through epithelial proliferation, angiogenesis, and immunomodulation. Mechanically isolated SVF preserves the 3D architecture of the extracellular matrix (ECM) and the original vasculature. The ECM facilitates cell–cell and cell–matrix interactions and functions as a reservoir for growth factors and cytokines as well as a release system for paracrine factors. In addition, ECM supports integrin-mediated binding of cells and functions as a scaffold to guide cell proliferation, migration and survival. This supports cell retention at the implementation site [51]. In contrast, enzymatic digestion disrupts the ECM, including all cell–cell interactions, and yields a single-cell suspension lacking this biological scaffold. Clinical outcome differences between mechanical and enzymatic isolation remain undetermined but are theoretically in favor of mechanically isolated SVF [52].

Observational studies evaluating fat grafting as an add-on to surgery reported high healing rates in both entities, and thus seem to be a promising add-on in the surgical treatment of perianal fistula [19,47]. This was supported by the recent conference abstract of Laureti et al., in which fat grafting in CD patients + fistulectomy was compared to fistulectomy only in a randomized trial. At 6-month follow-up, combined healing rates of 56.6% vs. 18.2% were reported. This is the first RCT performed on fat grafting in CD patients and shows promising results [53]. However, the precise mechanisms and importance of patient and/or product characteristics remain unclear. Tencerova et al. described cellular differences between healed perianal fistula and non-healed patients treated with cultured autologous adipose-derived mesenchymal stromal cells. Although no differences in cell yield were observed, increased CD49a (related to higher regenerative capacities), a lower expression of the inflammatory genes IL1B and NFKB, and a trend in increased expression of the anti-inflammatory IL10 gene were observed in the patient group with healed fistulae [54]. These findings indicate that product characteristics could be an important factor in treatment success and should be considered for systematic assessment.

The rationale for using PRP as an add-on to fistula surgery is grounded in its biological mechanism. PRP contains growth factors that stimulate fibroblast proliferation and angiogenesis, both essential processes in wound healing, including in perianal fistulas. Calcium chloride and thrombin syringes have been described to activate PRP; however, it remains unclear whether this influenced outcomes [55]. Despite its potential therapeutic effects as an add-on to surgery for the treatment of perianal fistulae, three out of four RCTs in cryptoglandular PAF did not report any superiority of PRP over standard care [23,30,42]. In addition to the activation method, the platelet concentration seems to be important for PRP efficacy, as shown by the meta-analysis by Bense et al. reporting a correlation between the concentration and clinical outcomes in knee osteoarthritis [56,57]. The association between platelet count and outcomes has not yet been described in perianal fistulae. In addition, none of the included studies using PRP or PRP combined with SVF reported on the platelet count of the injected product [25,28,31,37]. The only RCT reporting superiority of PRP over standard care was performed by Madbouly et al., reporting significantly higher healing rates in favor of PRP at one month of follow-up [34]. A comparable finding was reported by Ascanelli et al., who performed an RCT in cryptoglandular fistula, investigating fat grafting. Their results showed significantly higher healing rates at one- and three-month follow-up among patients receiving fat grafting as compared to surgical intervention alone. However, by six months, healing rates were comparable to those in the control group. This suggests that fat grafting or PRP could enhance early healing. These findings are consistent with a study in which SVF was applied to wounds following mammoplasty. Improved wound healing was observed up to 6 months compared with untreated wounds; however, this effect diminished by 12 months [58]. Such accelerated early healing may reduce work- and productivity losses by enabling an earlier return to work in this young population.

In the studies by Bak et al., treatment with PRS, a combined product of PRP and SVF, was applied as an add-on to closure of the internal fistula orifice. Mechanistically, combining PRP with SVF (containing ASCs), may act synergistically: PRP provides multiple growth factors, including VEGF, IGF-1 and TGF-β, which can enhance ASC proliferation, viability, and stimulate the production of paracrine factors relevant for tissue repair [59,60,61,62]. This may be valuable in an ischemic environment such as a perianal fistula [63]. Yet, the precise mechanisms remain partly understood, and differences between in vitro and in vivo environments complicate mechanistic interpretation [64].

Reported complication rates in the included studies were low and mostly related to the surgical intervention itself and not as a consequence of the addition of adipose-derived therapy or PRP, suggesting safety of adipose-derived therapies and PRP on the short-term. Maintaining continence after fistula surgery is a major clinical priority. However, less than 30% of included studies assessed continence as a secondary outcome, highlighting a limitation of the current evidence. Repeated interventions have been associated with scar tissue formation, higher rates of incontinence, and reduced healing potential, emphasizing the need for therapies that minimize fibrosis. Adipose-derived therapies might have these potentials as they have been shown to reduce fibrosis and increase elastic fibers in scar tissue. [65,66]. This effect may translate into lower incontinence rates, although this has not yet been properly assessed. Also, the effect of and the need for reinterventions to achieve complete closure should be further explored. It seems that refractory or complex perianal fistula may benefit from repeated interventions to achieve closure rates between 60 and 90% [29,30,32,40,45].

Heterogeneity in patient selection (refractory disease, prior surgical intervention, the use of biologicals), fistula complexity (number of tracts, low vs. high fistulae), type of surgical intervention (closure of internal opening, LIFT, TAFR), add-on therapy, timing of follow-up (3, 6, or 12 months) and outcome definitions (clinical vs. radiologic) complicate comparison of results across studies. As previously described by Pelly et al., heterogeneity among trials in perianal fistulas remains a common challenge [67]. This heterogeneity prevents quantitative synthesis, restricts the ability to draw firm conclusions and limits comparability and generalizability of the included studies. It may suggest that add-on therapies could be more effective in selected subgroups, such as patients with complex or refractory fistulas.

Greater uniformity in study design and inclusion criteria is required to generate reliable and reproducible evidence. The TOpClass consortium has defined a classification system for CD-related PAF and recommends restricting trial inclusion to patients with a type 2a fistula (“patients with symptomatic fistulae suitable for combined medical and surgical closure or repair [including seton removal] and whose goal is fistula closure”). The classification does not include an objective definition, and some variation is therefore inevitable; it does, however, offer guidance for patient selection [68]. Beyond classification, the importance of adopting a combined endpoint, namely clinical and radiologic healing, is underscored by the PISA-II trial which reported that complete radiologic healing (a completely hypointense, fibrotic fistula tract) is associated with sustained remission, long-term healing and no need for reinterventions in CD-related PAF. Less than forty percent of the studies included in this review used a radiologic endpoint. To objectify future research, we recommend radiologic assessment of fistula healing using scoring indices such as the modified Van Assche index and/or the MAGNIFI-CD for CD-related PAF [7,69,70]. Recurrence, particularly in CD-related fistulas, can occur years after initial treatment. Longer follow-up is therefore needed to accurately assess durability. As only five studies reported long-term outcomes, currently available short-term recurrence rates may be optimistic, especially given the mostly clinical healing definitions. Building on current guidelines, we emphasize the need for randomized trials comparing surgery with and without autologous therapies in the treatment of perianal fistula [71,72].

### Limitations

The main limitation of this review is the heterogeneity among the included studies. Autologous therapies are inherently variable, directly driving heterogeneity, and the lack of standardized clinical guidelines or protocols, further contributes to variations across studies. Only a small number of RCT’s were available, which differed in disease entity, complexity and surgical intervention, limiting the comparability of their outcomes. Additionally, radiologic healing was not consistently assessed across studies, preventing conclusions on structural tissue regeneration. Our findings might be influenced by publication bias, as abstracts and unpublished studies were excluded, potentially leading to an overestimation of the true effect.

By focusing on the direct applicability of autologous regenerative therapies this review adds insights into the current literature, which to date mostly focuses on allogeneic therapies or stromal cells with the need of laboratory processing. Other recent advances further highlight progress in regenerative bioengineering, including decellularized extracellular matrix (dECM)-based materials, exosomes and 3D bioprinted scaffolds. In parallel, emerging 3D MRI-based modeling approaches facilitate improved anatomical visualization, treatment evaluation and preoperative planning, enabling more precise surgical strategies [73].

## 5. Conclusions

The available evidence on autologous intraoperative adipose-derived regenerative therapies and PRP is limited by differences in patient populations, fistula complexity, interventions and outcome definitions. The majority of studies are low quality and lack control groups. While these therapies may support healing, firm conclusions regarding the effectiveness cannot be drawn. Reported healing rates, recurrence rates and complication rates underscore the therapeutic potential and provide a rationale for further well-designed studies with long-term follow-up.

## Figures and Tables

**Table 1:** Study characteristics.

Author	Study Design	Disease Type (*n*)	Total (*n*)	Therapy	Follow-Up (Months)	Control	Control (*n*)	Female *n* (%)	Age Mean Years	BMI (Mean kg/m^2^)	Active Smoking *n* (%)	Extent/Branches	Previous Therapy	Repeated Injections	Work Up Prior Treatment	Concomitant Anti-TNF-α	Concomitant Immunomodulator
Ascanelli et al. 2021 [19]	RCT	CGrPAF	116	ASCs	15 (9–29)	Surgery	58	45 (39)	50	26	NR	Horsehoe *n* = 2 (3,5%)	Seton *n* = 38 (65.5%) Surgery *n* = 33 (56.9%)	Yes	-	NA	NA
Bak et al. 2025 [45]	C1G	CDrPAF	25	tSVF + PRP	44	NA	NA	14 (66)	35 (25–40)	NR	4 (16)	Infralevatoric extensions *n* = 40 (40%) Supralevatoric extensions *n* = 6 6 (25%) Horseshoe configuration *n* = 9 (38%)	Fistulotomy *n* = 2 (8%) Drainage + seton *n* = 9 (38%) Drainage + drain *n* = 15 (63%) Seton *n* = 17 (68%) Laser treatment *n* = 1 (4%)	yes	-	ADA *n* = 2 (4%) IFX *n* = 5 (20%), VEDO *n* = 1 (4%), UST *n* = 2 (8%) Combination *n* = 10 (40%)	1 (4%)
Bak et al. 2024 [20]	RCS	CGrPAF	219	PRP	12	TAFR	131	65 (30)	46 ± 11	NR	NR	Multiple side tracts *n* = 126 (61,7%) 1 EO *n* = 182 (86.7%) 2 EO *n* = 27 (12.9%) 3 EO *n* = 1 (0.5%)	Seton drainage, endorectal advancement flap or LIFT in *n* = 84 (95.5%)	No	6 weeks seton	-	NA
Bak et al. 2025 [41]	C1G	CGrPAF	43	tSVF + PRP	50 (42–61)	NA	NA	13 (30)	46 (12.0)	NR	NR	No side tracts *n* = 20 (47%) 1 side tract *n* = 11 (26%) >2 side tracts *n* = 12 (27%)	Prior fistula surgery *n* = 41 (95%) Prior fistula surgery aimed at closure *n* = 22 (56%)	Yes	-	NR	NR
Bananzadeh et al. 2025 [42]	RCT	CGrPAF	85	PRP	12	LIFT	45	36 (42%)	44	26.0	19 (22)	Single tract	History of perianal surgery N = 17 vs. *n* = 24	NR	NR	NA	NA
Dalby et al. 2023 [21]	RCS	CGrPAF	77	FAT G	6	NA	NA	49 (64)	47 (44–50)	27.8 (26.5–29.0)	7 (9)	Single tract	Previous closure attempt 30% *n* = 23 LIFT *n* = 9 (39.1%) TAFR *n* = 2 (8.7%) fistulotomy *n* = 7 (30.4%)	Yes	-	-	-
De la Portilla et al. 2017 [24]	C1G	CGrPAF	36	PRP	12	NA	NA	8 (22)	51 ± 12.8	30. ± 6.5	11 (31)	>1 tracts *n* = 11	NR	No	12 weeks seton	-	NR
De la Portilla et al. 2019 [23]	RCT	CGrPAF	56	PRP	12	FG ^α^	24	11 (10.5)	50 ± 13.22	30.3 ± 6.7	31 (56)	NR	NR	No	-	-	NR
De la Portilla et al. 2020 [22]	C1G	CDrPAF	29	PRP	24	NA	NA	10 (34.5)	38 (18–68)	25.8 (20.9–32.6)	10 (34.5)	1 EO *n* = 16 (55.2%) 2 EO *n* = 7 (24.1%) 3 EO *n* = 6 (20.7%)	NR	No	-	IFX: *n* = 6 (20.7%) ADA: *n* = 8 (27.6%)	Yes, 15 (51.7%)
Dige et al. 2019 [25]	C1G	CDrPAF	21	FAT G	6	NA	NA	15 (71)	NR	NR	NR	NR	Refractory to seton or curettage.	Yes	seton	IFX *n* = 12 (57.1%) ADA *n* = 3 (14.3%)	AZA = 9 MCP = 1 + MTX AZA = 2 VEDO = 1
Göttgens et al. 2014 [26]	RCS	CGrPAF	25	PRP	12	NA	NA	8 (32)	49 (23–75)	27.6 [19.1–35.3]	5 (20)	NR	0 operations *n* = 12 (48%) 1 operation *n* = 6 (24.5%) 2 operations *n* = 3 (12%) > 2 operations *n* = 4 (16%)	Yes	3 months seton	NR	NR
Göttgens et al. 2015 [27]	C1G	CDrPAF	10	PRP	12	NA	NA	7 (70)	47 (30–67)	25.7 [21.1–32.4]	4 (40)	NR	No intervention *n* = 5 (50%) 2 interventions *n* = 1 (10%) 3 interventions *n* = 3 (30%)	Yes	3 months seton	Anti-TNF-α *n* = 7 (70%)	*n* = 5 (50%)
Guillo et al. 2022 [28]	C1G	CDrPAF	10	cSVF + FAT G	27 [4–77]	NA	NA	4 (40)	37 ± 12.8	24.4 ± 5	Active 3 (30)	1 tract *n* = 4 (40%) 2 tracts *n* = 5 (50%) >2 tracts *n* = 1 (10%)	1 anti-TNF *n* = 10 (100%) 2 anti-TNF *n* = 8 (80% Vedolizumab *n* = 3 (30%) Deviating ileostomy *n* = 2 (20%) Drainage with setons *n* = 10 (100%)	No	seton	Anti-TNF-α *n* = 7 (70%) VEDO *n* = 3 (30%)	NA
Hermann et al. 2021 [29]	C2G	CGrPAF	50	PRP	12	PDP ^α^	25	12 (48)	31 (29–39)	NR	NR	NR	Excision, fistulotomy, TAFR and LIFT in PRP *n* = 19 (76%)	Yes	seton	NA	NA
Hermann et al. 2022 [30]	RCT	CGrPAF	96	PRP	12	47	47	26 (53)	31 (29–39)	NR	NR	NR	Excision: *n* = 14 (29%) Fistulotomy: *n* = 9 (18%) TAFR: *n* = 4 (8%) LIFT: *n* = 2 (4%)	Yes	seton	NA	NA
Herreros et al. 2019 [31]	OCS	CGrPAF24 (57%) Other 10	42	cSVF	20 [6–48]	NA	NA	21 (47)	45 (24–69)	NR	NR	1 tract *n* = 37 (88%) 2 tracts *n* = 3 (7%) 4 tracts *n* = 2 (5%)	Drainage *n* = 2 Drainage + seton *n* = 31 Endoanal flap *n* = 2 Drainage + ASC’s *n* = 5 Endoanal flap & flap/plug = 1	Yes	-	NR	NR
Huang et al. 2023 [32]	C1G	CGrPAF *n* = 18 (34.6%) CDrPAF *n* = 34 (65.4%)	52	FAT G	12 [1–59]	NA	NA	30 (58)	39 ± 11.9	26.5 ± 5.6	NR	NR	Seton placement (81.5%) LIFT or flap (29.6%)	Yes	6–12 weeks Seton	Medication per procedure (*n* = 81): UST *n* = 29 (35.8%) IFX *n* = 10 (12.3%) ADA *n* = 8 (9.9%) VEDO *n* = 4 (4.9%) Other *n* = 2 (2.5%) None *n* = 3 (3.7%)	AZA *n* = 18 (22.2%) MTX *n* = 10 (12.3%) MCP *n* = 1 (1.2%)
Jeong et al. 2023 [33]	RCS	CGrPAF	22	cSVF	15.5 (3–36)	NA	NA	2 (9)	48 (28–71)	NR	NR	NR	2 procedures: *n* = 3 (13.6%) 3 procedures: *n* = 17 (77.2%)	No	-	No	NA
Laureti et al. 2025 [40]	C1G	CDrPAF	15	FAT G	80 (78–94)	NA	NA	8 (53)	40 ± 11.7 (22–58)	NR	NR	Posterior horseshoe *n* = 5 Branching *n* = 6	Ileocecal resection/subtotal colectomy *n* = 6 (40%) Diverting ileostomy *n* = 35(20%) Fistulectomy + seton *n* = 15 (100%) TAFR *n* = 12 (80% Plug *n* = 3 (20%)	No	-	IFX *n* = 7 (46.7%) ADA *n* = 10 (66.7%) VEDO *n* = 3 (20%)	Corticosteroids *n* = 1, MZ *n* = 4 AZA *n* = 2
Madbouly et al. 2021 [34]	RCT	CGrPAF	98	PRP	12	LIFT	49	33 (35.9)	44 ± 13.6	26 [23–32]	9 (18)	>1 EO *n* = 5 (5%) Horseshoe tract *n* = 9 (9%)	hemorrhoidectomy *n* = 7 sphincterotomy *n* = 4	No	-	-	NR
Maya et al. 2024 [43]	C1G	CGrPAF (*n* = 9) CDrPAF (*n* = 8)	17	PRP	6	NA	NA	9 (53%)	47 (2–63)	NR	4 (24)	NR	I&D *n* = 9 (52.9%) Pressfit placement *n* = 7 (41.1%) Advancement flap *n* = 6 (35.3%) Permacol placement *n* = 4 (23.5%) LIFT *n* = 3 (17.6%) Stem cells placement *n* = 2 (11.7%) Endosponge *n* = 1 (5.8%) Sphincteroplasty *n* = 1 (5.8%)	Yes	4–6 weeks seton	NR	NR
Naldini et al. 2017 [35]	C2G	CGrPAF	19	SVF	9 [3–12]	NA	NA	12 (63.2)	48 (28–77)	NR	NR	Multiple tracts were excluded	NR	No	4–6 weeks seton	NR	NR
Niknami et al. 2022 [36]	C1G	CGrPAF	24	PRP	12	NA	NA	8 (33.3)	41 ± 11.99 (22–60)	NR	NR	NR	NR	No	-	NR	NR
Potenza et al. 2024 [44]	C1G	CDrPAF	10	tSVF	6	NA	NA	3 (30)	33.6 ± 13.4	NR	NR	>1 fistula *n* = 2	Partial fistulectomy with seton (*n* = 8)	No	4 weeks seton	IFX *n* = 5 (50%) ADA *n* = 2 (20%) Vedo *n* = 2 (20%) UST *n* = 1 (10%)	NR
Reyes Diaz et al. 2025 [46]	C1G	CGrPAF	166	PRP	60	NA	NA	52 (31.3)	51.4 ± 13.2	29.5 ± 5.3	NR	Mean No. of tracts 1.21 ± 0.45	≤3 interventions 105 (63.6) > 3 interventions 40 (24.2)	Yes	Seton if abscess	-	-
SØrensen et al. 2022 [37]	C1G	CDrPAF	12	FAT G + cSVF	12	NA	NA	9 (75)	33 (22- 51)	27.9 [20.07–37.03]	3 (25)	2 EO *n* = 2	6-month medical treatment	No	-	Anti-TNF-α *n* = 6	AZA
Stroumza et al. 2017 [38]	PCS	CGrPAF	11	FAT G	6	NA	NA	4 (44)	58 (39–66)	NR	4 (44)	NR	Seton, fistulotomy and/or fistulectomy (*n* = 3) All recurrent disease	No	4–6 weeks seton	NR	NR
Topal et al. 2019 [39]	RCS	CGrPAF	10	cSVF	9	NA	NA	2 (20)	47 ± 13.1 (37–69)	28.3 ± 4.79 [22.5–37.3]	NR	NR	Seton *n* = 2 (20%), LIFT *n* = 2 (20%), Fistulotomy *n* = 1 (10%)	No	-	-	NR

Where indicated, values are mean [standard deviation] or mean (range). C1G = cohort study (1 group, pre- + postoperative); C2G = cohort study (2 groups, pre- + postoperative) prospective cohort study, RCS = retrospective cohort study, OCS = Observational cohort study, RCT = randomized control trial. CDrPAF = Crohn’s disease-related perianal fistula, CGrPAF = Cryptoglandular perianal fistula. NR = not reported. NA = not applicable.. PDP porcine derived paste. TAFR = Transanal flap repair, FAT G = fat graft, EO = external opening, tSVF = tissue stromal vascular fraction, cSVF = cellular stromal vascular fraction, PRP = platelet-rich plasma, ASC = adipose-derived stromal cells, LIFT = ligation of the intersphincteric fistula tract. FG = fibrin glue. ADA = adalimumab, AZA = azathioprine, IFX = infliximab, VEDO = vedoluzimap UST = ustekinumab, MCP = Mercaptopurine, MTX = methotrexate, MZ = mesalazine ^α^ = no region of interest, excluded from further analysis.

**Table 2:** Outcomes and definition of healing in fistulas treated with regenerative therapies.

Author	Closure Definition		Intervention	Closure Rates *n* (%)		Reintervention	Recurrence	Secondary Outcomes	Complications	
	Clinical	Radiological		Clinical	Radiological				Minor	Major
Ascanelli et al. 2021 [19]	EO closure, no symptoms and drainage	NR	CF + surgery vs. surgery *	*n* = 37 (63.8%) vs. *n* = 9 (15.5%) *p* < 0.001 at 4 weeks *n* = 51 (87.9%) vs. *n* = 37 (63.8%) *p* < 0.001 at 3 months *n* = 50 (86.2%) vs. *n* = 47(81%) (*p* = 0.47) at 6 months	*n* = 50 (86%) vs. *n* = 35 (60%) at 3-month follow-up	*n* = 7 (87.5%) vs. *n* = 8 (80%) *p* = 0.802	*n* = 8 (13.7%) vs. *n* = 11 at 6 months (*p* = 0.477) *n* = 2 (3.4%) vs. *n* = 0 (0%) at 12 months (*p* = 0.496)	No difference in Cleveland Clinical Florida and Fecal Incontinence Score at 6 months	Abdominal pain/hematoma: *n* = 11 (18.9%) Abscess *n* = 1 (1.7%) vs. *n* = 4 (6.9%) *n* = 0.170 Hemorrhoids *n* 11 (18.9) Bleeding *n* = 1 (1.7%)	None
Bak et al. 2025 [45]	EO closure, no symptoms and no drainage	Complete absence of fluid containing fistula tracts	tSVF + PRP + CcIO	Clinical response *n* = 17 (68%) Complete clinical closure at 12 months *n* = 13 (52%) Complete clinical closure at 3 years FU *n* = 22 (88%)	Complete at 12 months *n* = 9 (38%) Complete long-term *n* = 18 (75%)	*n* = 13 *n* = 6 (46%) second injection *n* = 4 (31%) third injection *n* = 3 (23%) fourth injection	NR		Wound infection *n* = 1: (4%) Perianal hemorrhage: *n* = 2 (8%).	None
Bak et al. 2024 [20]	EO closure No fluid discharge	NA	PRP + TAFR vs. TAFR	Overall healing PRP + TAFR *n* = 65 (73.9%) vs. TAFR *n* = 101 (77.1%) (*p* = 0.58	NA	*n* = 7 vs. *n* = 11	*n* = 3 (6.3) vs. *n* = 2 (2.9%) with a mean follow-up of 6.8 (SD 3.7) years.	NA	After TAFR: Bleeding *n* = 9 (4.2%) treated conservatively	After TAFR: Abscess *n* = 7 (3.3%) wherefore I&D. flap dehiscence *n* = 1 (0.5%), needing additional flap repair
Bak et al. 2025 [41]	EO and IO closure, no fluid discharge	Total absence of hyperintense signal.	tSVF + PRP + TAFR	*n* = 39 (91%) at 12 months	*n* = 35 (81%)	*n* = 5 second injection of tSVF + PRP *n* = 1 third infection of tSVF + PRP	*n* = 4 (9%) after clinical closure	MRI	None	
Bananzadeh et al. 2025 [42]	EO closure No fluid discharge	NA	PRP + LIFT vs. LIFT	*n* = 36 (90%) vs. *n* = 35 (77.8%) (*p* = 0.153)	NA	NR	*n* = 4 vs. *n* = 5 (*p* = 0.735)	No difference in healing at 12-month follow-up Perianal pain (VAS) at 1-week follow-up 2 vs. 4 (*p* < 0.001) Time to healing (days) 28 vs. 65 (*p* < 0.001)	No complications vs. Perianal infection *n* = 5 Perianal abscess *n* = 1	None
Dalby et al. 2023 [21]	EO closure, no palpable IO, no symptoms of discharge.	Absence of collections > 2 cm at the location of the former fistula	MFAT + CcIO	*n* = 28 (37%) at 3 months *n* = 39 (51%) at 6 months	*n* = 37 second injection -> 27% (*n* = 10) healing at 6 months	NR	NR	NA	Hematoma: 21%, Proctalgia: 18%, Temporary minor incontinence: 2.6%, Anal infection: 5.3%, Pain requiring opioid: 5.3%, urinary retention <24 h resolved: 2.6%	Bleeding: 1.8% Anal abscess: 1.8% Infected hematoma: 0.9%
De la Portilla et al. 2017 [24]	Complete epithelialization EO, no suppuration	NA	PRP + PPP clot + CcIO	*n* = 7 (33.3%) Partial: 8 (38.1%) 24 weeks 15 patients at 48 weeks postop Complete 6 (40%)	NA	NR	NR	Visual analog score improvement significant after 6 months of treatment (*p* = 0.0278) and 1 years after treatment (*p* = 0.0438) Improvement in Wexner scores 6 months (*p* = 0.0550) and 1 year (*p* = 0.0195)	Diarrhea (due to AB) *n* = 4, Fecal incontinence *n* = 1, urgency def *n* = 1	Urine tract infection, Scrotal + prostate abcess and rectourethral fistula (*n* = 1)
De la Portilla et al. 2019 [23]	EO closure and epithelialization, no symptoms of discharge Partial healing: No fully epithelialization, no suppurative discharge, one fistula closed in case multiples fistula opening	No fistula opening, no abscess cavity identifiable	PRP + PPP clot + CcIO	*n* = 15 (48.4%) vs *n* = 10 (41.7%)	TRUS: *n* = 14 (48.3%) vs. *n* = 9(42.9%) Correlated with the clinical findings.	NR	*n* = 18 (32.1%) total 31.3% vs. 33.3%	Wexner score 0 (range 0–6.75) Greater decrease in median pain scores in PRP treated group No difference in Quality of life domains	Proctalgia *n* = 1 Perianal abscess *n* = 1 (spontaneously drained)	
De la Portilla et al. 2020 [22]	EO closure and epithelialization, no discharge Partial healing: No fully epithelialization, no suppurative discharge, one fistula closed in case multiples fistula opening	NA	PRP + PPP clot + CcIO	Complete healing *n* = 12 (33.3%) and partial healing *n* = 4 (11.1%) at 24 weeks (*n* = 21) 48 weeks (*n* = 15) Complete healing *n* = 6 (40%) and partial healing *n* = 6 (40%) at 48 weeks (*n* = 15)		NR	NR	No difference in PDAI (range 2–9) vs. (range 1–8) at final visit	Pain requiring additional analgesia: *n* = 2, perianal swelling responsive to AB: *n* = 1	Abscesses requiring drainage and withdrawal from the study *n* = 2
Dige et al. 2019 [25]	EO closure, no discharge, no palpable IO	No signs of a fluid-conducting tract at the location of the former fistula	MFAT + CcIO	*n* = 9 (43%)	*n* = 8 (38.1%)	*n* = 9 second injection and 2 healed *n* = 4 third injection and 1 healed	*n* = 2 (%)	NA	Proctalgia OR pain after liposuction: *n* = 4, Urinary retention, *n* = 1, Liposuction bleeding *n* = 1	Labia majora abscess *n* = 1
Göttgens et al. 2014 [26]	EO closure, no symptoms	NA	PRP + TAFR	100% in 3 months Primary closure rate *n* = 21 (84%) Secondary closure rate *n* = 23 (92%)	NA	*n* = 2 (8%) second injection N = 1 (4%) colostomy	*n* = 4	No difference in Vaizey score	None	Abscess *n* = 1 (4%)
Göttgens et al. 2015 [27]	NR	NR	PRP + CcIO	*n* = 8 (80%) at 3 months *n* = 9 (90%) >3 months 70% at 1 year	NA	*n* = 5	*n* = 1 (10%) *n* = 2 repeated injections (1 no healing -> deviating colostomy and 1 no closure)	NA	None	Abscess *n* = 1 (10%)
Guillo et al. 2022 [28]	NR	The MAGNIFI-CD score ranges from 0 (inactive disease) to 25 (severe disease)	cSVF + microfat + CcIO	Combined remission *n* = 6 (60%) and Clinical response *n* = 2 (20%) at 1 year Combined remission *n* = 7 (70%) at 3 years	mean MAGNIFI-CD (17.8 ± 4.9 to 14.7 ± 4.7) at 3 years	*n* = 1 ileocecal resection for stenosis *n* = 1 deviating colostomy	*n* = 1 (10%) after 3-years follow-up	PDAI: 7.3 ± 2.7 vs. 3.4 ± 4.3 vs. 7.2 ± 5.2 at baseline, 1 year, and 3 years, respectively SIBDQ: 43.4 ± 11.9 vs. 47.0 ± 11.4 vs. 49.0 ± 13.2 at baseline, 1 year, and 3 years, respectively	Pain at harvesting site *n* = 4 (40%), local inflammation *n* = 4 (40%), cutaneous reaction *n* = 1 (10%)	Perianal abscesses *n* = 3 (30%) wherefore seton (*n* = 3) and antibiotics (*n* = 2).
Hermann et al. 2021 [29]	No discharge EO	Closure confirmation by TRUS	PRP + CcIO	*n* = 16 (64%) at 12 months.	NR	3–5 times repeated injections	*n* = 3 (12%)	No significant difference in Wexner score at 12 months	NR	NR
Hermann et al. 2022 [30]	NR	NA	PRP (only) vs. TAFR	PRP *n* = 35 (71.43%) vs. TAFR *n* = 27 (57.45%) (*p* = 0.152)	NA	*n* = 14 first injection *n* = 9 second injection *n* = 12 third injection	No recurrence in both groups at 24 months	No significant difference between PRP and TAFR for complete closure, Wexner score	None	TAFR: postoperative bleeding *n* = 2
Herreros et al. 2019 [31]	No suppuration EO At 6 months	NA	cSVF + CcIO OR TAFR	Overall, 53% (after 1 injection) CD-related 40% Crypto 69.2%	NA	*n* = 3 second injection -> 3 healed in CD	NA	NA	None	None
Huang et al. 2023 [32]	EO closure, no drainage, no pain, no seton	NA	CF + CcIO	*n* = 29 (64.4%) after 1 injection CDrPAF *n* = 17 (50.0%) vs. CGrPAF *n* = 12 (70.6%) (*p* = 0.23)	NA	Multiple injections in CDrPAF *n* = 10 and CGrPAF *n* = 6, *n* = 7 (45.7%) closure	NR	NA	Bleeding: *n* = 1.	Postoperative abscess with surgical drainage *n* = 7.
Jeong et al. 2023 [33]	EO closure 50%, no suppuration from EO at 6 months	NA	cSVF + CF + CcIO	*n* = 19 (86%) 3 months *n* = 21 (95%) 9 months	NA	No	None	NA	None	None
Laureti et al. 2024 [40]	EO closure, no drainage, no symptoms, no seton	NA	MFAT + curettage	*n* = 14 (93.3%) at 24 months	*n* = 10 (66.7%) *n* = 13 (87%)	Second injection and TAFR *n* = 3 Third injection and TAFR *n* = 1	*n* = 1 (10%) at 6.7-years follow-up)	CRP, 1.1 ± 1.0 mg/dL at T24 vs. 1.7 ± 1.1 mg/dL at T0; ESR, 26.7 ± 12.8 mm at T24 vs. 19.3 ± 14.1 mm at T0; PDAI, 4.3 ± 2.8 at T24 vs. 7.5 ± 2.1 at T0). The IBDQ score increased (160.9 ± 57.8 at T24 vs. 143.1 ± 38.5 at T0), while SF-36 physical (PCS) and mental scores (MCS) were not significantly changed (PCS, 40.1 ± 10.8 at T24 vs. 41 ± 9.2 at T0; MCS: 39.1 ± 12 at T24 vs. 33.7 ± 11.3 at T0	Hematoma: *n* = 1	None
Madbouly et al. 2021 [34]	EO en IO closure, no symptoms of discharge or inflammation.	NA	LIFT + PRP vs. LIFT	LIFT + PRP: *n* = 38 (77.6%) vs. LIFT: *n* = 29 (59.2%) (*p* = 0.03)	NA	NR	LIFT-PRP: 4 (9.5%; 95% CI 4.3–22.4) vs. LIFT: 3 (9.3%; 95% CI6.6–23.6) p0.99 one-year follow-up.	Quality of life and level of happiness were significantly higher in LIFT-PRP compared to LIFT (*p* < 0.05). The median Wexner score in both groups was 0 (range, 0–5)with no significant difference. Pain VAS at d 1 3.6 ± 1.01 vs. 2.0 ± 1.1 *p* = 0.01 Pain VAS at d 7 2.7 ± 1.34 vs 1.2 ± 0.71 *p* = 0.01	Fecal impaction: *n* = 1.	Perianal abscess: *n* = 2
Maya et al. 2024 [43]	Absence of discharge	NA	PRP + CcIO	*n* = 9 (53%) CD *n* = 3 (38%) CG *n* = 6 (66%)	NA	*n* = 3	*n* = 5	NR		Abscess *n* = 1
Naldini et al. 2017 [35]	NR	NA	MFAT + CcIO	*n* = 14 (73.7%)	NA	NR	NR	NR	Hematoma: *n* = 3	Perianal abscess: *n* = 1
Niknami et al. 2022 [36]	EO and IO closure, no fluid discharge, >2 months	NA	PRP +CcIO	*n* = 16 (66.7%)	NA	NR	1m: *n* = 4 (16.3%), 6m: *n* = 2 (10%), 1y: *n* = 2 (11%)	NR	None	None
Potenza et al. 2024 [44]	Closure of EO, no drainage of fluid after finger compression	absence of perianal abscesses >3 mm or fluid in the tract of treated fistula	tSVF + CcIO	*n* = 8 (80%)	*n* = 5 (50%)	Second injection *n* = 1	*n* = 1 (10%) at 11 months	Quality of Life in healed patients at 6 months vs. baseline (8.3 vs. 7.0) (*p* = 0.05)	NR	NR
Reyes Diaz et al. 2025 [46]	Absence of suppuration as well as the complete epithelialization of the external fistulous orifice	NA	PRP + CcIO	*n* = 118 (70.9%) at 1 year FU	NA	NR	NR	Incontinence rates after 1 year *n* = 22 (14.3%)	Abscess *n* = 10 (6.1%) Bleeding *n* = 2 (1.2%)	
Sorensen et al. 2022 [37]	NR	NR	LA + cSVF + CcIO	*n* = 37 (82%)	*n* = 33 (89.2%)	NA	*n* = 3 at 12-month follow-up	More than 50% reduction in mean WFIC score reduced at 6-month follow-up (1.4) compared to baseline measurement (5.4)	None	None
Stroumza et al. 2017 [38]	NR	NR	AFG + CcIO	*n* = 8 (73%) at 6 months	*n* = 8 (73%) at 6 months	NA		NR	None	None
Topal et al. 2019 [39]	EO closure, no fluid discharge.	NA	ASC + CcIO	*n* = 7 (70%) at 9 months	NA	NR	*n* = 2 (perianal abscess)	NA	Hematome *n* = 2	Perianal abscess *n* = 2

NR = not reported. NA = not applicable.. ASC = adipose-derived stromal cells, AFG = adipose fat graft, CDrPAF = Crohn’s disease-related perianal fistula, CGrPAF = cryptoglandular related perianal fistula, CF = coleman fat, EO = external orifice, IO = internal orifice, IBDQ = Infammatory Bowel Disease Questionnaire, LIFT = ligation of interspfinctering fistula tract, TAFR = Transanal flap repair, MFAT = micrografted adipose tissue, PDAI = Perianal Crohn’s Disease Activity Index, TRUS = transrectal ultrasonografic, WFIC = Wexner Fecal Incontinence Score.* surgical interventions varied between fistulectomy with or without advanced flap, video assisted anal fistula treatment with or without advanced flap, LIFT.

## Data Availability

No new data were created or analyzed in this study. Data sharing is not applicable to this article.

## Supplementary Materials

The following supporting information can be downloaded at: https://www.mdpi.com/article/10.3390/bioengineering13040393/s1, Supplementary File S1: Search strategy, Supplementary Table S1: PRISMA, Supplementary Table S2a: Quality Assessment Table, Supplementary Table S2b: Risk of bias assessments, Supplementary Table S3: Treatment characteristics.
