# Supplementary material for: Intraoperative Autologous Adipose-Derived Therapies and PRP as Add-On in the Surgical Treatment of Cryptoglandular and Crohn’s Disease-Related Perianal Fistula—A Systematic Review
Source: Bioengineering (Basel). 2026 Mar 28;13(4):393. doi: 10.3390/bioengineering13040393 (PMC13113760; doi:10.3390/bioengineering13040393)
Supplement: Supplementary file 1 [file bioengineering-13-00393-s001.zip › bioengineering-4176519-supplementary.pdf]

Supplementary material

Supplementary file S1 Search strategy

Medline 433

(* Stem Cell Transplantation/ OR Mesenchymal Stem Cell Transplantation/ OR Mesenchymal Stem Cells/ OR * Stem Cells/ OR Platelet-Rich Plasma / OR Stromal Vascular Fraction/ OR (((mesenchym* OR autolog*) ADJ3 (cell*) ADJ3 (transplant* OR Therap*)) OR ((mesenchym* OR autolog*) ADJ3 (stem-cell* OR platelet-rich* OR fat)) OR ((thrombocyte* OR platelet*) ADJ3 rich ADJ3 (plasma* OR stroma*)) OR (stroma* ADJ3 vascul* ADJ3 fraction*) OR (adipose ADJ3 (stem-cell* OR stroma*-cell*)) OR fat-graft* OR lipograft*).ab,ti,kf. OR (stem-cell* OR strom*-cell* OR platelet-rich* OR autolog*).ti.) AND ((Fistula/ AND Inflammatory Bowel Diseases/) OR Cutaneous Fistula/ OR Digestive System Fistula/ OR (fistul* ADJ6 (crohn* OR inflammator*-bowel* OR gastr* OR intestin* OR digest* OR rectal* OR recto* OR perianal* OR anal* OR in-ano OR cryptogland* OR enterocutan* OR crypto-gland* OR entero* OR enteral*)).ab,ti,kf.) NOT (exp animals/ NOT humans/) NOT (case-report*).ti.

Embase 279

('stem cell transplantation'/mj OR 'mesenchymal stem cell transplantation'/exp OR 'mesenchymal stem cell'/exp OR 'mesenchymal stroma cell'/exp OR 'stem cell'/mj OR 'autologous stem cell transplantation'/exp OR 'thrombocyte rich plasma'/de OR 'stromal vascular fraction'/de OR 'adipose derived stem cell'/de OR 'fat graft'/de OR 'fat grafting'/de OR (((mesenchym* OR autolog*) NEAR/3 (cell*) NEAR/3 (transplant* OR Therap*)) OR ((mesenchym* OR autolog*) NEAR/3 (stem-cell* OR platelet-rich* OR fat)) OR ((thrombocyte* OR platelet*) NEAR/3 rich NEAR/3 (plasma* OR stroma*)) OR (stroma* NEAR/3 vascul* NEAR/3 fraction*) OR (adipose NEAR/3 (stem-cell* OR stroma*-cell*)) OR fat-graft* OR lipograft*):ab,ti,kw OR (stem-cell* OR strom*-cell* OR platelet-rich* OR autolog*):ti) AND ((fistula/de AND 'inflammatory bowel disease'/exp) OR 'skin fistula'/de OR 'digestive system fistula'/exp OR (fistul* NEAR/6 (crohn* OR inflammator*-bowel* OR gastr* OR intestin* OR digest* OR rectal* OR recto* OR perianal* OR anal* OR in-ano OR cryptogland* OR enterocutan* OR crypto-gland* OR entero* OR enteral*)):ab,ti,kw) NOT [conference abstract]/lim NOT ([animals]/lim NOT [humans]/lim) NOT (case-report*):ti

Web of science 134

(TS=(((mesenchym* OR autolog*) NEAR/2 (cell*) NEAR/2 (transplant* OR Therap*)) OR ((mesenchym* OR autolog*) NEAR/2 (stem-cell* OR platelet-rich* OR fat)) OR ((thrombocyte* OR platelet*) NEAR/2 rich NEAR/2 (plasma* OR stroma*)) OR (stroma* NEAR/2 vascul* NEAR/2 fraction*) OR (adipose NEAR/2 (stem-cell* OR stroma*-cell*)) OR fat-graft* OR lipograft*) OR TI=(stem-cell* OR strom*-cell* OR platelet-rich* OR autolog*)) AND TS=((fistul* NEAR/5 (crohn* OR inflammator*-bowel* OR gastr* OR intestin* OR digest* OR rectal* OR recto* OR perianal* OR anal* OR in-ano OR cryptogland* OR enterocutan* OR crypto-gland* OR entero* OR enteral*))) NOT TI=(case-report*) AND DT=(article) AND LA=(english)

CINAHL 0

(MH Mesenchymal Stem Cells OR MM Stem Cells OR MH Platelet-Rich Plasma OR MH Stromal Vascular Fraction OR TI(((mesenchym* OR autolog*) N2 (cell*) N2 (transplant* OR Therap*)) OR ((mesenchym* OR autolog*) N2 (stem-cell* OR platelet-rich* OR fat)) OR ((thrombocyte* OR platelet*) N2 rich N2 (plasma* OR stroma*)) OR (stroma* N2 vascul* N2 fraction*) OR (adipose N2 (stem-cell* OR stroma*-cell*)) OR fat-graft* OR lipograft*) OR AB(((mesenchym* OR autolog*) N2 (cell*) N2 (transplant* OR Therap*)) OR ((mesenchym* OR autolog*) N2 (stem-cell* OR platelet-rich* OR fat)) OR ((thrombocyte* OR platelet*) N2 rich N2 (plasma* OR stroma*)) OR (stroma* N2 vascul* N2 fraction*) OR (adipose N2 (stem-cell* OR stroma*-cell*)) OR fat-graft* OR lipograft*) OR TI(stem-cell* OR strom*-cell* OR platelet-rich* OR autolog*)) AND ((MH Fistula AND MH Inflammatory Bowel Diseases+) OR MH Cutaneous Fistula OR MH Digestive System Fistula OR TI(fistul* N5 (crohn* OR inflammator*-bowel* OR gastr* OR intestin* OR digest* OR rectal* OR recto* OR perianal* OR anal* OR in-ano OR cryptogland* OR enterocutan* OR crypto-gland* OR entero* OR enteral*)) OR AB(fistul* N5 (crohn* OR inflammator*-bowel* OR gastr* OR intestin* OR digest* OR rectal* OR recto* OR perianal* OR anal* OR in-ano OR cryptogland* OR enterocutan* OR crypto-gland* OR entero* OR enteral*))) NOT (MH animals+ NOT MH humans+) NOT TI(case-report*)

Supplementary Table S1 PRISMA

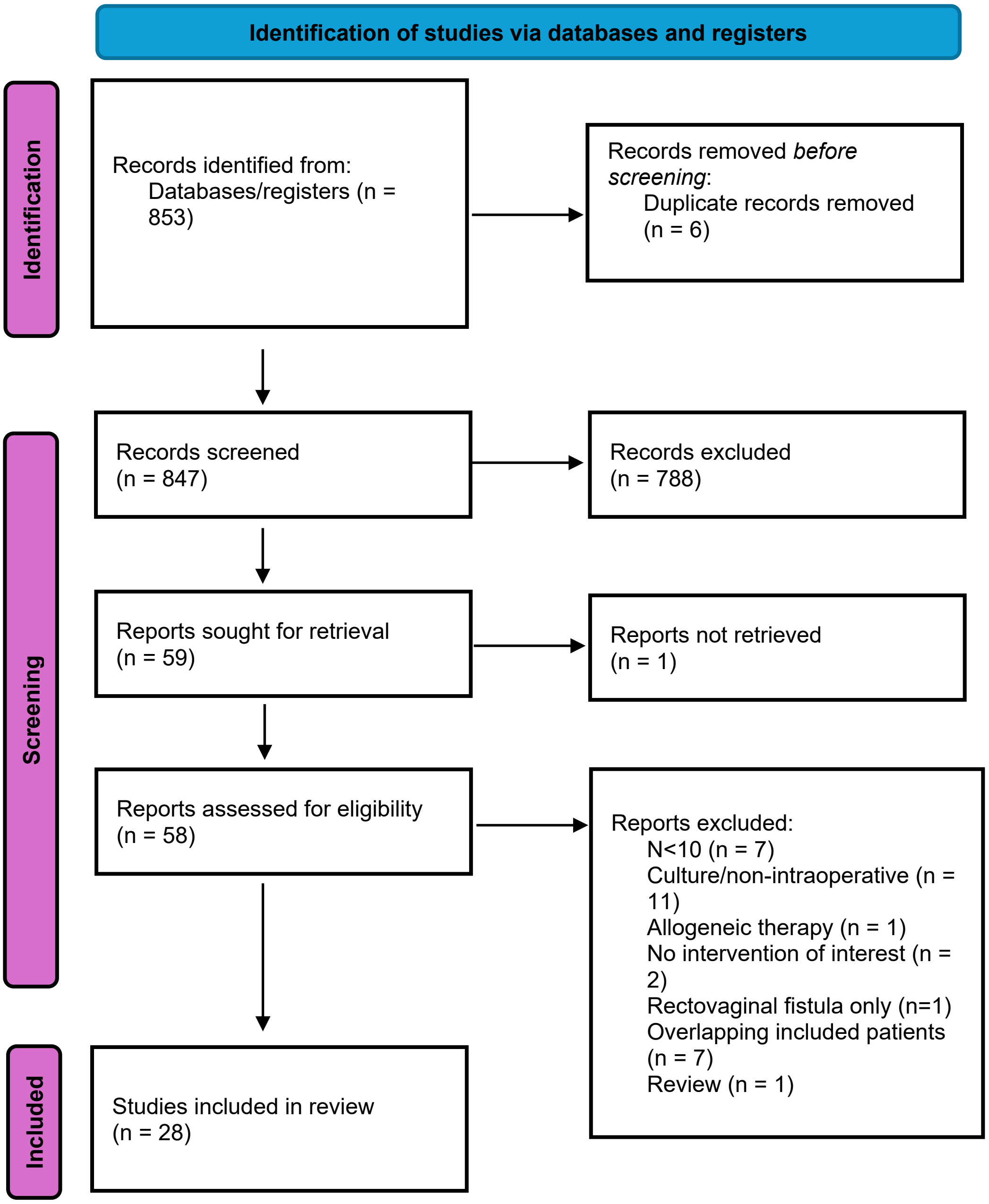

PRISMA 2020 flow diagram for new systematic reviews which included searches of databases and registers only

From: Page MJ, McKenzie JE, Bossuyt PM, Boutron I, Hoffmann TC, Mulrow CD, et al. The PRISMA 2020 statement: an updated guideline for reporting systematic reviews. BMJ 2021;372:n71. doi: 10.1136/bmj.n71

Supplementary Table S2a Quality Assessment Table

Study	Selection bias	Study Design	Confounders	Blinding	Data collection	Drop-outs	Global rating
Ascanelli et al. 2021 [19]	0	+	0	0	0	+	+
Bak et al. 2025 [45]	-	0	-	-	0	+	-
Bak et al. 2024 [20]	0	0	+	-	0	-	0
Bak et al. 2025 [41]	0	0	-	0	0	0	0
Bananzadeh et al. 2025 [42]	0	+	+	+	0	+	+
Dalby et al. 2023 [21]	0	0	-	-	+	+	-
De la Portilla et al. 2017 [24]	0	0	0	+	+	0	+
delaPortilla et al. 2019 [23]	0	0	0	0	0	+	+
delaPortilla et al. 2020 [22]	0	0	-	0	+	+	+
Dige et al. 2019 [25]	0	0	-	-	+	+	0
Göttgens et al. 2015 [27]	0	0	-	-	0	+	0
Göttgens et al. 2014 [26]	0	0	-	-	0	+	-
Guillo et al. 2022 [28]	0	0	-	-	0	0	-
Hermann et al. 2021 [29]	-	0	-	-	0	-	-
Hermann et al. 2022 [30]	0	+	-	-	0	-	-
Herreros et al. 2019 [31]	0	0	-	-	0	0	-
Huang et al. 2023 [32]	-	0	-	-	0	+	-
Jeong et al. 2024 [33]	-	0	-	-	-	-	-
Laureti et al. 2025 [40]	0	0	-	0	+	0	0
Madbouly et al. 2021 [34]	0	+	0	+	0	+	+
Maya et al. 2024 [43]	0	0	-	-	-	0	-
Naldini et al. 2018 [35]	0	0	-	-	-	-	-
Niknami et al. 2022 [36]	0	0	-	-	-	-	-
Potenza et al. 2024 [44]	0	0	-	-	0	-	-
Reyes Diaz et al. 2025 [46]	0	0	0	-	0	+	0
Sørensen et al. 2022 [37]	-	0	-	-	0	0	-
Stroumza et al. 2017 [38]	-	0	-	-	-	-	-
Topal et al. 2019 [39]	-	0	-	-	-	-	-
Weak, n (%)	7 (25%)	0 (0%)	21 (75%)	20 (71%)	5 (18%)	10 (36%)	16 (58%)
Moderate, n (%)	21 (75%)	24 (86%)	5 (18%)	5 (18%)	18 (64%)	7 (25%)	6 (21%)
Strong, n (%)	0 (%)	4 (14%)	2 (7%)	3 (11%)	5 (18%)	11 (39%)	6 (21%)

The totals at the bottom represent the distribution of how weak, moderate and strong each criterion is., +, strong, 0, moderate, –, weak

Supplementary Table S2b. Risk of bias assessments of the included randomized trials according to the revised tool to assess risk of bias in randomized trials (RoB 2).

	**Domain 1**	**Domain 2**	**Domain 3**	**Domain 4**	**Domain 5**	**Overall**
Acanelli et al.	Low	Low	Low	Low	Low	Low
Bananzadeh et al.	Low	High	Low	Some concerns	Some concerns	Some concerns
De la portilla et al. 2019	Low	Low	Low	Some concerns	Low	Low
Hermann et al.	Some concerns	Low	Low	Some concerns	Some concerns	Some concerns
Madbouly et al.	Low	Low	Low	Low	Low	Low

Author			Infiltration				Aspiration	Injection		Isolation procedure		Enzymatic (E) / Mechanical (M)	Procedure time mean/min	Volume or platelet count	Pre-/post treatment		Sample characterisation
Author	Intervention	Donor site	Infiltration	Lidocaine	Epinephrine	NaHCO3		Cannula	Site	System	Details				Pre	Post	
Ascanelli et al. 2021 [19]	FAT G + LIFT /VAAFT/TAFR	A	+	20 ml 2%	1ml	-	Coleman	22G	2cc PF 6cc IF	NA	Centrifugation 580 *g*/3 min	M	85 min	16ml (14-18)	NA	Analgesic	FACS, CFU, adipogenic and osteogenic differentiation assays, flowcytonmetry of expanded ASC's<br><br>Methods reported, data not shown[#]
Bak et al. 2025 [45]	tSVF + PRP + CcIO	A	+	20 ml 2%	5ml 1:1000	-	NR	NR	PF	ACP kit	FAT procedure: 960 *g*/2.5 min centrifugation, shuffling through a three-hole 1.4 mm connector and 960 *g*/2.5 min centrifugation<br><br>PRP: centrifugation 1500rpm/ 4 min, separation	M	70 (56-85)	SVF = 1 ml PRP = 5 ml	profyAB	-	NR
Bak et al. 2024 [20]	PRP + TAFR vs TAFR	-	-	-	-	-	-	NR	PF	ACP kit	FAT procedure: 960 *g*/2.5 min centrifugation, shuffling through a three-hole 1.4 mm connector and 960 *g*/2.5 min centrifugation<br><br>PRP: 1500rpm/ 4 min centrifugation, separation	M	NR	4-5ml	NA	NR	NR
Bak et al. 2025 [41]	tSVF + PRP + TAFR	A	+	20 ml 2%*	5ml 1:1000	-	NR	NR	PF	ACP kit	FAT procedure: 960 g/2.5 min centrifugation, shuffling through a three-hole1.4 mm connector and 960 g/2.5 min centrifugation<br><br>PRP:	M	>120-150	7ml	ProfAB	AB	NR

											Blood centrifugation (4 min, 1500 rpm)						
Bananzadeh et al. 2025 [42]	PRP + LIFT vs LIFT	PV	-	-	-	NR	-	NR	PF 5cc	NA	1500rpm/10min centrifugation, separation, collection upper layer, 4000rpm/5min, seperation	-	NR	5ml	ProfAB	Topical mupirocin, AB, psyllium powder 5 days	NR
Dalby et al. 2023 [21]	FAT G + CcIO	A, F, IT, B	+	-	1ml	-	3,5mm	1.2mm	PF	NA	1000rpm/3min centrifugation, aqueous fluid expelled, shuffling through Luer-Lok connector	M	79 (74-83)	61ml (40-85)	profyAB, analgesic,	AB	NR
De la Portilla et al. 2017 [24]	PRP + PPP cloth + CcIO	PV	-	-	-	NR	-	NR	PF + IF	NA	1800 rpm/8min, addition 10% CaCI2, endogenous activation, separation	-	NR	NR	profyAB,	Analgesic AB	NR
De la Portilla et al. 2019 [23]	PRP + PPP cloth + CcIO	PV	-	-	-	NR	-	NR	PF + IF	NA	Anticoagulant, 160g/6 min centrifugation, separation, collection middle layer, addition 10% CaCl,	-	26.3 (+-10) vs 31(+-13.1)	NR	profAB	Analgesic AB	NR
De la Portilla et al. 2020 [22]	PRP + PPP cloth + CcIO	PV	-	-	-	NR	-	NR	PF + IF	NA	Anticoagulant, 160g/6 min centrifugation, separation, collection middle layer, addition 10% CaCl	-	NR	8-10ml	profAB	Analgesics AB	NR
Dige et al. 2019 [25]	FAT G + CcIO	A	+	40ml	1ml	-	3.5mm	2.5mm blunt Coleman/ 1.2mm	PF	NA	1000 rpm/3min centrifugation, shuffling through Luer-Lok connector	M	NR	46ml (18-104)	profAB	Analgesics AB	Cell yield: 4.1 x10 ^{5} cells/mL<br>Cell viability: 99%<br>FACS:<br>Stromal cells (CD44/CD90/CD105) = 49%-57%, stromal cells (CD44+/CD271+) = 8%,endothelial cells (CD31+/CD34+) = 20%, monocytes/macrophages (CD14+/CD45+) = 1%

Göttgens et al. 2014 [26]	PRP cloth + TAFR	PV	-	-	-	-	-	NR	IF	GPS-III	Thrombin coated syringe to activate PRP	-	30	NR	NR	NR	NR
Göttgens et al. 2015 [27]	PRP cloth + TAFR	PV	-	-	-	-	-	NR	IF	GPS-III	Thrombin coated syringe to activate PRP	-	NR	NR	NR	NR	NR
Guillo et al. 2022 [28]	cSVF + FAT G + CclO	NR	+	+	+	-	Khouri	25/21G	PF	CYT	NR	E	NR	10.8ml (5-17)	NR	AB	Cell viability: 22.8 x 10^6 (10.9-47.8 x 10^6)
Hermann et al. 2021 [29]	PRP + CclO vs PDP	NR	-	-	-	NR	-	NR	PF	Xerthra	NR	-	NR	NR	NR	NR	NR
Hermann et al. 2022 [30]	PRP vs TAFR	NR	-	-	-	NR	-	22/23G	PF	Xerthra	NR	-	NR	1-3ml	NR	NR	NR
Herreros et al. 2019 [31]	cSVF + TAFR/CclO	NR	NR	NR	NR	-	NR	NR	PF	CYT GID ADSC	NR	E	NR	NR	NR	NR	Cell yield: 43.9 x10^6 cells (3-210x10^6)
Huang et al. 2023 [32]	FAT G + LIFT/TAFR/CclO	A, F, H,	+	50ml 1%	1ml 1:1000	-	3mm	NR	PF	NA	Centrifugation 20 min	M	NR	4-6ml	profAB	AB Stool softener	NR
Jeong et al. 2023 [33]	cSVF + FAT G + CclO	A,F, B, IT	NR	NR	NR	-	NR	1.5G	PF	Enzymatic closed system kit<br><br>Mix 1:10 (SVF:scaffold)	SVF: Digestion collagenase and shaken 30 min, centrifugation 1500g/3 min, washing<br><br>fat scaffold: lipoaspirate, centrifugation 1240g/ 3min	E	NR	SVF 5.8 ml(2-7) Scaffold 22.5ml (10-32)	NR	NR	NR
Laureti et al. 2025 [40]	FAT G + fistulotomy	A, IT	+	40 ml 2%	1 ml 1:1000	-	13G	21G	PF	LIPO	lipoaspirate, washing with saline, filtration (reduction and micronization with 5 stainless balls)	E	NR	20ml	NR	NR	NR
Madbouly et al. 2021 [34]	PRP + LIFT	NR	-	-	-	NR	-	NR	PF	GPS	NR, no activation of PRP	-	NR	4ml PRP solution + 1ml PRP gel	profyAB	AB	NR
Maya et al. 2024 [43]	PRP + CclO	NR	-	-	-	NR	-	NA	IF	Obsidian RFT	NR	-	NR	NR	NR	Analgesic	NR
Naldini et al. 2017 [35]	cSVF + CclO	A	+	20 ml 1%	1 ml	-	13G	22G	PF	LIPO	NR	E	55(50-70)	16ml ±2.9 (12-22)	NA	NR	NR

Niknami et al. 2022 [36]	PRP + CcIO	NR	-	-	-	NR	-	NR	PF + IF	NA	Anticoagulant, 850g centrifugation, seperation, 1200g centrifugation lower layer	-	NR	12ml	NR	NR	NR
Potenza et al. 2024 [44]	tSVF + CcIO	H	NR	NR	NR	NR	2.1mm	22G	PF	NT	Mechanical emulsification microfat through filters (2.4 mm and 1.2 mm, centrifugation 3000rpm, 3min, 600/400 µm filtering.	M	73 (43-119)	NR	NR	NR	NR
Reyes Diaz et al. 2025 [46]	PRP + CcIO	PV	NR	NR	NR	-	-	-	PF	NA	Centrifuge at 1800 8min. Addition 10% CaCl.	-	38 ±3.5	-	-	Analgesics Diet	NR
Stroumza et al. 2017 [38]	FAT G + CcIO	A	+	-	1 ml :1000	-	3mm	NR	PF	PURE =2, Revolve= 7 Decantation= 2	Washing and filtration in bag	M	42 (28-75)	21ml (10-30)	-	AB	NR
Topal et al. 2019 [39]	cSVF + CcIO	A	+	20 ml 1%	1ml	-	13G	NR	PF	LIPO	NR	M	45±7 (40-80)	10ml (2/cm tract)	NR	1-2 days hospital stay	NR

Supplementary Table S3. Treatment characteristics

Where indicated, values are mean [standard deviation] or mean(range).
A = abdomen, B = buttocks, F = Flank, IT = inner thigh, H = hips, PV = peripheral vene, NR= not reported, + = applicable, - = not applicable, PF = perifistular, IF = intrafistular, ACP kit= autologous condition plasma kit: Double syringe (Arthrex©), ADSC= ADSC system (Lyposmol Biotech, Zaragoza, Spain), FAT G= fat graft, CYT= Cytori celution 800 IV Celution 800/IV system (Lorem Cytori, San Diego, California, USA), CcIO = Curettage and closure of the internal opening, FACS= Fluorescence-activated cell sorting, GID= GID system (Karam Medical, Barcelona, Spain), GPS = gravitational platelet separation, (Cell Factor Technologies, Biomet, Warsaw, Indiana, USA), LIPO= Lipogems (lipogems, International Spa, Milan, Italy), NT = Nanotranfer, Tulip Medical PDP = Porcine derived paste, PPP = platelet poor plasma, PURE= PureGraftTM (Cytori Therapeutics, San Diego, California, USA), Revolve= RevolveTM (LifeCell, Bridgewater, New Jersey, USA), Xerthra= Xerthra PRPKit (Biovico, Poland)
*xylocaine was used
#different types of sample characterisation used is shown in a reference article, however no data of the used cells is reported in the study.
